# Effects of postoperative radiotherapy on cardiovascular-pulmonary disease mortality in patients with stage IIIA-N2 resected NSCLC: analysis of the SEER database

**DOI:** 10.1186/s13014-021-01912-4

**Published:** 2021-09-20

**Authors:** Xia Wang, Jiaqi Song, Jie Long, Zhimin Zeng, Anwen Liu

**Affiliations:** 1 Department of Oncology, The Second Affiliated Hospital of Nanchang University, No. 1 Minde Street, Nanchang, 330000 Jiangxi Province People’s Republic of China; 2 Jiangxi Key Laboratory of Clinical Translational Cancer Research, Nanchang, 330000 Jiangxi Province People’s Republic of China; 3 Radiation Induced Heart Damage Institute of Nanchang University, Nanchang, 330000 Jiangxi Province People’s Republic of China

**Keywords:** Postoperative radiotherapy, Non-small cell lung cancer, Stage IIIA-N2, Cardiovascular-pulmonary disease mortality, SEER, Propensity score matching

## Abstract

**Background:**

The role of postoperative radiotherapy (PORT) in cardiovascular-pulmonary disease mortality in patients with stage IIIA-N2 resected non-small cell lung cancer (NSCLC) remains uncertain. The purpose of this population-based analysis was to explore the effect of PORT on cardiovascular-pulmonary disease mortality in these patients.

**Methods:**

Patients aged ≥ 18 years with stage IIIA-N2 resected NSCLC were identified in the Surveillance, Epidemiology, and End Results (SEER) database from 2004 to 2015 and were grouped according to the use of PORT. Propensity score matching (PSM) was used to account for differences in baseline characteristics between the Non-PORT and PORT groups. The cumulative risk for cardiovascular-pulmonary disease death was estimated using the cumulative incidence curve. Competing risk regression was used to run univariate and multivariate analyses to evaluate risk factors.

**Results:**

A total of 3981 patients were included in the study population. Among them, 1446 patients received PORT, and 2535 did not. A total of 1380 patients remained in each group after PSM, and the baseline characteristics were not significantly different between the two groups. The cumulative incidence of cardiovascular-pulmonary mortality was 10.93% in the Non-PORT group compared with 9.85% in the PORT group. There was no significant difference in the cumulative risk between the two groups (HR 1.07, 95% CI 0.77–1.48, *p* = 0.703). Multivariate analysis indicated that PORT had no significant impact on increased risk, with an HR of 1.18 (*p* = 0.377).

**Conclusions:**

No significant differences between the PORT and Non-PORT groups were found in cardiovascular-pulmonary-specific modalities in this study. Further studies are required to validate these results. This study highlights the importance of long-term surveillance for NSCLC patients.

## Background

Lung cancer is the leading cause of cancer-related deaths worldwide, and non-small cell lung cancer (NSCLC) accounts for the vast majority of lung cancer cases [1, 2]. Early-stage NSCLC is best managed with complete surgical resection [3]. Despite curative-intent surgical resection, tumor recurrence and metastasis are major causes of death for patients with locally advanced NSCLC [4–6]. Therefore, surgery plus multidisciplinary sequential therapy continues to be the backbone of treatment with curative intent among patients with stage IIIA resected NSCLC [5, 7–9].

Previous studies have shown that postoperative radiotherapy (PORT) in patients with stage IIIA-N2 NSCLC reduces the risk of local recurrence and thus is an appealing means of improving outcomes in NSCLC patients [10, 11], but whether PORT can bring overall survival (OS) benefits to those patients remains controversial [9–12]. Several retrospective studies and meta-analyses have shown the survival benefits of PORT [5, 10, 11, 13–15]. However, recent multi-institutional randomized phase III trials (Lung ART and PORT-C) indicated that PORT failed to improve disease‐free survival and OS [9, 12]. In the Lung ART study, the incidence of grade 3–5 late cardiopulmonary toxicity was 20% versus 7.7%, and the cardiopulmonary specific mortality was 16.2% versus 2% in the PORT versus Non‐PORT cohort, respectively [9]. The survival benefit may be counterbalanced by radiotherapy (RT)‐induced cardiopulmonary-specific death [9].

Thoracic RT increases the risk of cardiovascular-pulmonary disease during or after therapy, such as ischemic heart disease, arterial disease, pericardial disease, vascular and metabolic issues, conduction disorders, pneumonitis and pulmonary fibrosis, chronic pulmonary insufficiency, and cor pulmonale, and resulting in increased mortality [6, 16–20]. Radiation-associated cardiovascular-pulmonary events and deaths have been thoroughly documented in long-term survivors of breast cancer and Hodgkin’s lymphoma [18, 21–24]. However, the data regarding RT‐associated cardiovascular-pulmonary specific death in patients with NSCLC are limited [6]. Currently, there are no large datasets available with PORT and cardiovascular-pulmonary specific mortality in patients with stage IIIA-N2 NSCLC.

Therefore, we conducted a propensity-matched retrospective study to investigate the effect of PORT on cardiovascular-pulmonary related death in patients with resected stage IIIA-N2 NSCLC using the Surveillance, Epidemiology, and End Results (SEER) database.

## Patients and methods

### Data sources

The data were downloaded from the SEER database using SEER∗STAT software (version 8.3.9). The SEER program of the National Cancer Institute in the United States collects data from 18 population-based registered cancer institutes, covering approximately 28% of cancer cases in the United States.

### Study population and definition

**Fig. 1:**
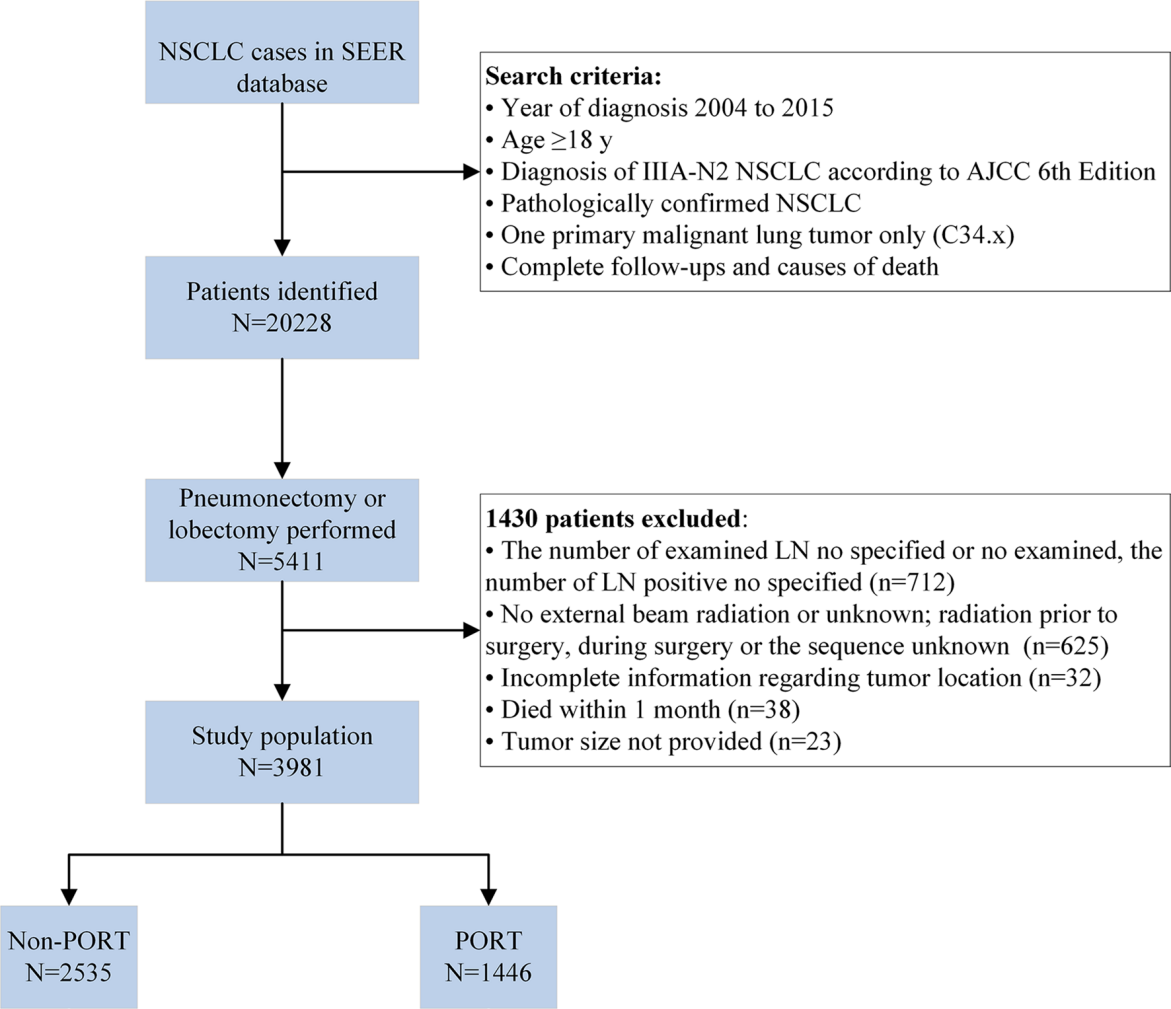
Flow chart of the screened patients. NSCLC non-small cell lung cancer, SEER Surveillance, Epidemiology, and End Results, AJCC American Joint Committee on cancer, LN lymph nodes, PORT postoperative radiotherapy

We extracted the data of patients with NSCLC registered from 2004 to 2015. The eligibility criteria included the following: (1) age older than 18 years; (2) pathologically confirmed NSCLC (histologic types were selected as adenocarcinoma [codes: 8140, 8250–8255, 8260, 8310, 8323, 8333, 8480, 8481, 8490, 8550, 8570, 8574], squamous cell carcinoma [codes: 8052, 8070–8074, 8083, 8084], and other NSCLC [codes: 8012, 8013, 8022, 8031–8033, 8035, 8046, 8050, 8082, 8123, 8200, 8201, 8430, 8560, 8980]; (3) diagnosis of stage IIIA-N2 NSCLC according to AJCC 6th Edition; (4) one primary malignant lung tumor only (C34.x); (5) previous lobectomy or pneumonectomy (SEER Surgery of Primary Site Codes range were 30–48 [lobectomy] and 55–70 [pneumonectomy]); (6) complete follow-ups and causes of death; and (7) complete record of RT information (received PORT or did not receive any RT). Major exclusion criteria included patients with incomplete registration information required by this study and those who died within 1 month. Patients were divided into Non-PORT and PORT groups according to whether they underwent PORT. Details of the patient selection process are shown in Fig. 1.

Variables extracted from the SEER database included age, sex, race, year of diagnosis, laterality (right and left), primary site, tumor size, histology code and behavior, pathologic grade, surgical procedure (lobectomy and pneumonectomy), T stage, the number of lymph nodes (LN) examined, the number of positive LN, radiation sequence with surgery, chemotherapy, survival months, vital status recode, cause of death, and SEER cause-specific death classification. The LN ratio was defined as the ratio of the number of positive LN to the number of examined LN.

Follow-up time was defined as the period between the initial diagnosis of lung cancer and events defined as death, the last follow-up, or the end of follow-up time (December 31, 2017), whichever came first. Cardiovascular-pulmonary disease-related death was calculated using coded causes of death from cardiovascular diseases (including heart diseases, hypertension without heart disease, cerebrovascular diseases, atherosclerosis, aortic aneurysm/dissection, or other diseases of arteries, arterioles, and capillaries) or pulmonary diseases (including chronic obstructive pulmonary disease and allied cond, or pneumonia and influenza) [22, 25, 26].

### Statistical analysis

Data are given as median (range) or n (%). Clinicopathological characteristics were compared between groups using Fisher’s exact test for categorical variables and the two-sample t-test or Mann-Whitney U-test for continuous variables, as appropriate. Propensity score matching (PSM) with 0.01 matching tolerance was used to balance baseline characteristics between Non-PORT and PORT groups. The cumulative risk for cardiovascular-pulmonary disease death was estimated using the cumulative incidence curve. Competing risk regression (Fine and Gray method) was used to run univariate and multivariate analyses to evaluate risk factors, considering noncardiovascular-pulmonary death as a competing event [27]. Differences were considered statistically significant at *p* values < 0.05. R software packages (http://www.R-project.org, The R Foundation) and Empower Stats software (http://www.empowerstats.com, X&Y Solutions, Inc., Boston, MA) were used to analyze all data.

## Results

### Patient characteristics

A total of 3981 patients fulfilled our inclusion criteria and were included in the study population. A flow chart of the selection process is shown in Fig. 1. Among them, 1446 (36.32%) patients received PORT. The proportions of patients receiving PORT differed by age, year of diagnosis, primary site, surgical procedure, metastatic LN, positive LN ratio, receipt of chemotherapy, and follow-up time (Table 1). No significant differences in sex, race, laterality, tumor size, histology, grade, T stage, or LN examined were seen between those who received PORT and those who did not. Using PSM at a ratio of 1:1, 1380 patients remained in each group. There were no significant differences in the clinicopathological patient characteristics between the Non-PORT and PORT groups after PSM, as shown in Table 1.

**Table 1:** The baseline clinical characteristics of enrolled patients with stage IIIA-N2 NSCLC before and after PSM

Clinical parameters	Before PSM			After PSM		
	Non-PORT (N = 2535)	PORT (N = 1446)	*P*-value	Non-PORT (N = 1380)	PORT (N = 1380)	*P*-value
Age, years (range)	67 (22–90)	64 (19–88)	< 0.001*	65 (22–89)	65 (28–88)	0.674
*Sex*			0.942			0.469
Male	1253 (49.43%)	713 (49.31%)		669 (48.48%)	688 (49.86%)	
Female	1282 (50.57%)	733 (50.69%)		711 (51.52%)	692 (50.14%)	
*Race*			0.585			0.591
Black	256 (10.10%)	141 (9.75%)		152 (11.01%)	136 (9.86%)	
White	2051 (80.91%)	1161 (80.29%)		1091 (79.06%)	1109 (80.36%)	
Others or unknown	228 (8.99%)	144 (9.96%)		137 (9.93%)	135 (9.78%)	
*Year of diagnosis*			< 0.001*			0.360
2004–2009	1370 (54.04%)	645 (44.61%)		653 (47.32%)	629 (45.58%)	
2010–2015	1165 (45.96%)	801 (55.39%)		727 (52.68%)	751 (54.42%)	
*Laterality*			0.154			0.378
Right	1373 (54.16%)	817 (56.50%)		760 (55.07%)	783 (56.74%)	
Left	1162 (45.84%)	629 (43.50%)		620 (44.93%)	597 (43.26%)	
*Primary site*			0.021*			0.766
Main bronchus	30 (1.18%)	16 (1.11%)		18 (1.30%)	15 (1.09%)	
Upper lobe	1415 (55.82%)	869 (60.10%)		793 (57.46%)	822 (59.57%)	
Middle lobe	113 (4.46%)	74 (5.12%)		67 (4.86%)	71 (5.14%)	
Lower lobe,	911 (35.94%)	463 (32.02%)		476 (34.49%)	448 (32.46%)	
Overlapping lesion of lung	66 (2.60%)	24 (1.66%)		26 (1.88%)	24 (1.74%)	
Tumor size, mm (range)	35 (1–195)	35 (5–180)	0.325	35 (1–190)	34 (5–180)	0.584
*Histologic type*			0.136			0.876
Adenocarcinoma	1622 (63.98%)	970 (67.08%)		920 (66.67%)	912 (66.09%)	
Squamous cell carcinoma	597 (23.55%)	307 (21.23%)		292 (21.16%)	303 (21.96%)	
Others	316 (12.47%)	169 (11.69%)		168 (12.17%)	165 (11.96%)	
*Grade*			0.604			0.855
Well differentiated	126 (4.97%)	58 (4.01%)		64 (4.64%)	55 (3.99%)	
Moderately differentiated	1062 (41.89%)	603 (41.70%)		586 (42.46%)	574 (41.59%)	
Poorly differentiated	1132 (44.65%)	655 (45.30%)		611 (44.28%)	625 (45.29%)	
Undifferentiated; anaplastic	64 (2.52%)	34 (2.35%)		34 (2.46%)	33 (2.39%)	
Unknown	151 (5.96%)	96 (6.64%)		85 (6.16%)	93 (6.74%)	
*Surgical procedure*			< 0.001*			0.239
Lobectomy	2229 (87.93%)	1324 (91.56%)		1242 (90.00%)	1260 (91.30%)	
Pneumonectomy	306 (12.07%)	122 (8.44%)		138 (10.00%)	120 (8.70%)	
*T stage (sixth edition)*			0.075			0.122
T1	697 (27.50%)	411 (28.42%)		391 (28.33%)	391 (28.33%)	
T2	1667 (65.76%)	912 (63.07%)		899 (65.14%)	871 (63.12%)	
T3	171 (6.75%)	123 (8.51%)		90 (6.52%)	118 (8.55%)	
LN examined (range)	11 (1–90)	11 (1–79)	0.812	11 (1–90)	11 (1–79)	0.595
*Metastatic LN*			< 0.001*			0.644
> 4	630 (24.85%)	450 (31.12%)		392 (28.41%)	403 (29.20%)	
<=4	1905 (75.15%)	996 (68.88%)		988 (71.59%)	977 (70.80%)	
*Positive LN ratio (%)*			< 0.001*			0.386
> 50	488 (19.25%)	376 (26.00%)		301 (21.81%)	320 (23.19%)	
<=50	2047 (80.75%)	1070 (74.00%)		1079 (78.19%)	1060 (76.81%)	
*Chemotherapy*			< 0.001*			0.673
No	1028 (40.55%)	151 (10.44%)		158 (11.45%)	151 (10.94%)	
Yes	1507 (59.45%)	1295 (89.56%)		1222 (88.55%)	1229 (89.06%)	
Median FU, months (range)	27 (1–154)	28 (1–154)	0.010*	29 (1–154)	28.5 (1–154)	0.852

LN, lymph node; FU, follow-up time; PSM, propensity score-matching; PORT, postoperative radiotherapy

*P < 0.05 was considered significant and marked in bold. Data represent as median (range) or n (%)

### Cumulative incidence of cardiovascular-pulmonary disease-related death

**Fig. 2:**
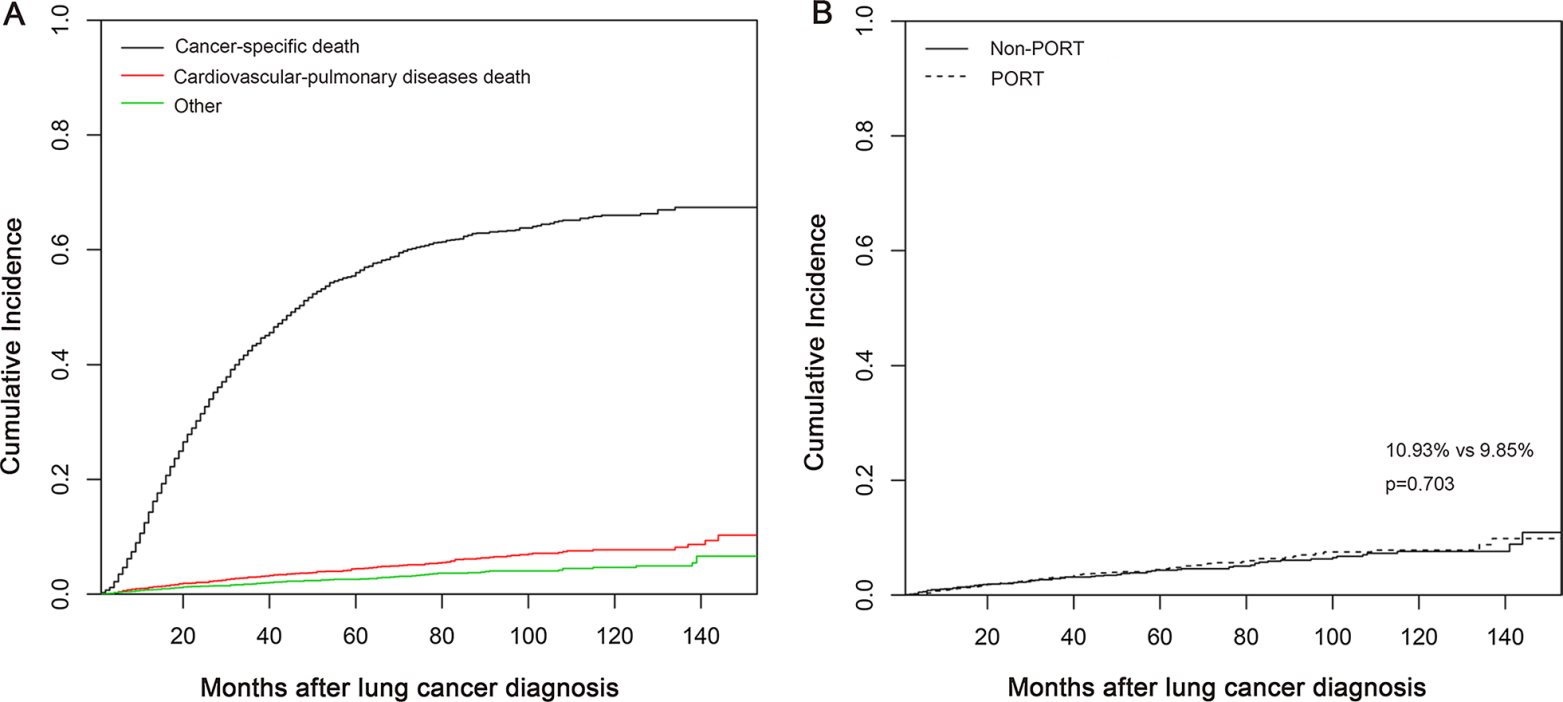
Cumulative incidence curves: (**A**) for cancer-, cardiovascular-pulmonary- and other cause-specific mortality; (**B**) for cardiovascular-pulmonary disease-related death by PORT use. PORT postoperative radiotherapy

A total of 1708 patients (61.88%) succumbed to primary NSCLC, cardiovascular-pulmonary disease, or deaths due to other causes in the period of 2004 to 2015. The cumulative incidence curve for all causes of death is shown in Fig. 2A. Primary NSCLC remained the leading cause of death for our cohort after PSM, followed by cardiovascular-pulmonary diseases, with cumulative incidence rates of 67.37 and 10.28%, respectively.

A total of 143 patients (5.18%) died of cardiovascular-pulmonary diseases. Among them, 70 and 73 patients died of cardiovascular-pulmonary causes in the Non-PORT and PORT groups, respectively. The cumulative incidence curve for cardiovascular-pulmonary death is shown in Fig. 2B. The cumulative incidence of cardiovascular-pulmonary mortality was 10.93% in the Non-PORT group compared with 9.85% in the PORT group. There was no significant difference in the cumulative risk between the two groups (hazard ratio [HR] 1.07, 95% confidence interval [CI] 0.77–1.48, *p* = 0.703).

### Risk factors for cardiovascular-pulmonary diseases death

**Fig. 3:**
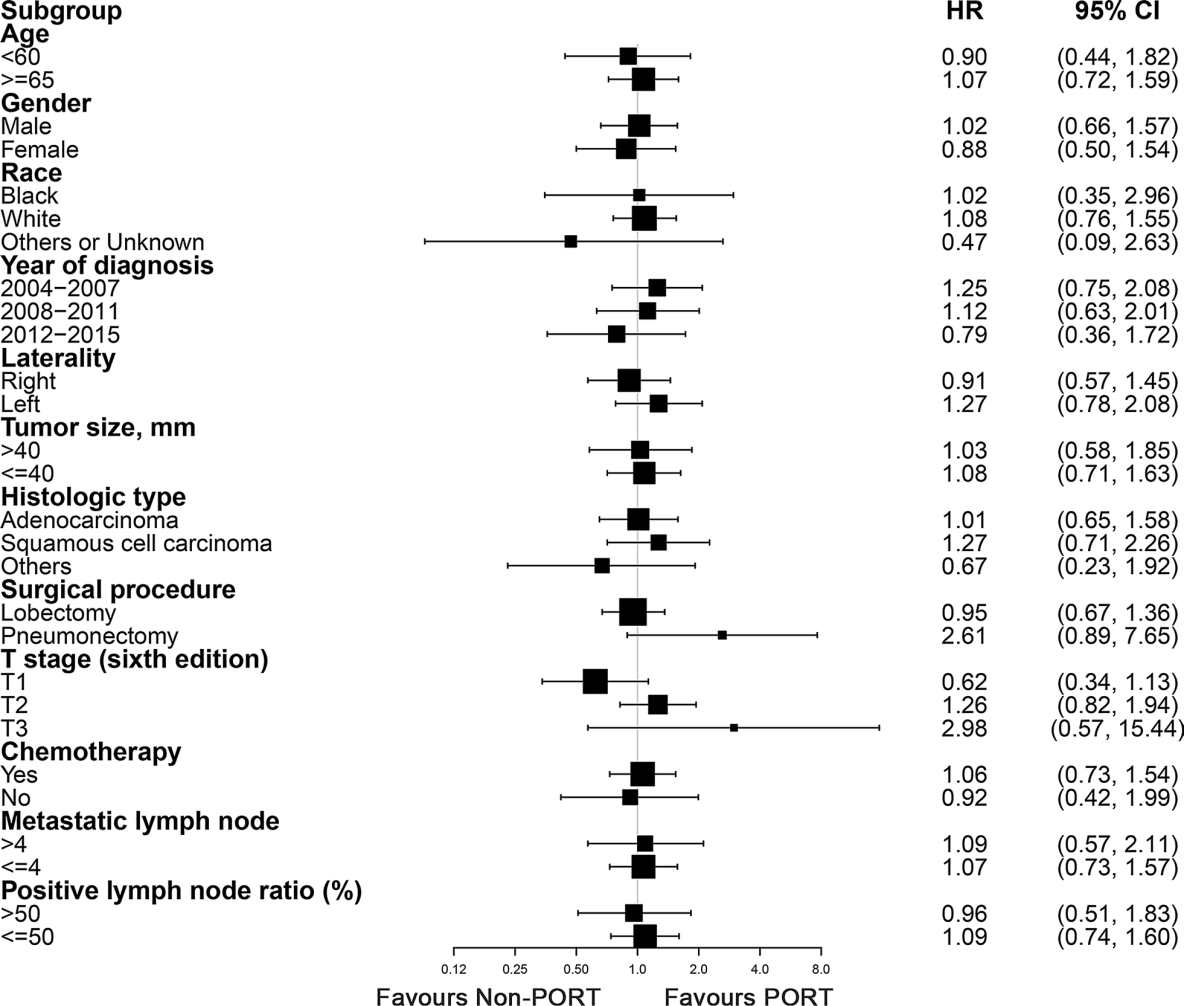
Subgroup analyses of cardiovascular-pulmonary disease death by PORT use. HR hazard ratio, CI confidence interval, PORT postoperative radiotherapy

Univariate Fine-Gray hazard model analysis revealed that cardiovascular-pulmonary disease-related mortality was significantly associated with age ≥ 60 (*p* = 0.020), male sex (*p* = 0.004), year of diagnosis between 2004 and 2007, squamous cell carcinoma (*p* < 0.001), and no receipt of chemotherapy (*p* = 0.001) (Table 2). Multivariate analysis showed that male sex (*p* = 0.026), year of diagnosis between 2004 and 2007, and squamous cell carcinoma pathologic type (*p* = 0.010) were independent risk factors for cardiovascular-pulmonary disease-related death (Table 2). We analyzed the impact of PORT on cardiovascular-pulmonary specific mortality, and the univariate and multivariate results suggested that PORT had no significant impact on increased risk (univariate: HR = 1.07, 95% CI 0.77–1.48, *p* = 0.703; multivariate: HR = 1.18, 95% CI 0.82–1.71, *p* = 0.377) (Table 2). Furthermore, subgroup analyses revealed similar results to the primary analyses, and patients treated with PORT were not associated with an increased risk of cardiovascular-pulmonary specific mortality compared to those who were not treated with PORT. The HRs and 95% CIs of the different subgroups are listed in Fig. 3.

**Table 2:** Univariate and multivariate analyses of cardiovascular-pulmonary disease-related mortality using a Fine-Gray hazard model

Variable name	HR	95% CI for HR		*p*-value
* Univariate analysis (N = 2760)*				
Age, years ( > = 60 vs. < 60)	1.60	1.08	2.38	0.020*
Sex (Male vs. Female)	1.65	1.18	2.32	0.004*
*Race*				
Black	Reference			
White	1.15	0.66	2.00	0.618
Others or unknown	0.46	0.17	1.20	0.111
*Year of diagnosis*				
2004–2007	Reference			
2008–2011	0.67	0.46	0.98	0.037*
2012–2015	0.31	0.19	0.48	< 0.001*
Laterality (Left vs. Right)	1.18	0.85	1.64	0.330
*Primary site*				
Main bronchus	Reference			
Upper lobe	0.85	0.21	3.48	0.820
Middle lobe	0.47	0.09	2.59	0.385
Lower lobe,	0.96	0.23	3.96	0.950
Overlapping lesion of lung	0.68	0.09	4.85	0.696
Tumor size, mm ( < = 40 vs. > 40)	1.09	0.77	1.54	0.628
*Histologic type*				
Adenocarcinoma	Reference			
Squamous cell carcinoma	1.93	1.34	2.78	< 0.001*
Others	1.03	0.59	1.79	0.919
*Grade*				
Well differentiated	Reference			
Moderately differentiated	0.87	0.37	2.03	0.750
Poorly differentiated	1.13	0.49	2.60	0.778
Undifferentiated; anaplastic	1.16	0.32	4.11	0.823
Unknown	0.85	0.29	2.47	0.771
Surgical procedure (Pneumonectomy vs. Lobectomy)	1.26	0.75	2.12	0.374
*T stage (sixth edition)*				
T1	Reference			
T2	0.81	0.57	1.15	0.244
T3	0.71	0.35	1.44	0.342
Chemotherapy (No vs. Yes)	2.04	1.35	3.08	0.001*
Metastatic lymph node ( < = 4 vs. > 4)	1.18	0.81	1.70	0.395
Positive lymph node ratio (%) ( < = 50 vs. > 50)	0.79	0.54	1.14	0.204
PORT (Yes vs. No)	1.07	0.77	1.48	0.703
*Multivariate analysis (N = 2760)*				
Age, years ( > = 60 vs. < 60)	1.36	0.87	2.14	0.179
Sex (Male vs. Female)	1.57	1.06	2.32	0.026*
*Year of diagnosis*				
2004–2007	Reference			
2008–2011	0.60	0.40	0.91	0.015*
2012–2015	0.29	0.18	0.49	**< 0.001**
*Histologic type*				
Adenocarcinoma	Reference			
Squamous cell carcinoma	1.70	1.13	2.54	0.010*
Others	0.84	0.46	1.53	0.569
Chemotherapy (No vs. Yes)	1.41	0.88	2.25	0.154
PORT (Yes vs. No)	1.18	0.82	1.71	0.377

Model adjusted for multivariate analysis: Age, Year of diagnosis, Sex, Histologic type, Chemotherapy, PORT.

HR, hazard ratio; CI, confidence interval; PORT, postoperative radiotherapy

*P < 0.05 was considered significant and marked in bold

## Discussion

Cardiovascular-pulmonary disease is the leading nonrecurrence cause of death in NSCLC patients [6]. The cardiovascular system and lungs are among the organs that are most severely affected by RT injury, resulting in increased morbidity and mortality [6, 16, 18]. The mechanisms of RT-related cardiovascular disease include endothelial dysfunction, altered vascular tone, hemostatic imbalance, and inflammatory activation [16]. Previous preclinical research found that radiation triggers lung injury by initiating a cascade of inflammatory reactions, with capillary leaks and alveolar and interstitial exudate, which later organize into collagen [18, 19]. Damage can even occur at least 10 years after RT [28].

The present work provides important insights into the risk of cardiovascular-pulmonary disease mortality by PORT in patients with stage IIIA-N2 resected NSCLC based on the SEER database. Our analysis of data from 2760 patients after PSM demonstrated that PORT among those patients was not associated with a higher risk of cardiovascular-pulmonary disease-related death (HR = 1.18, 95% CI 0.82–1.71, *p* = 0.377). The same conclusions were obtained in the subgroup analyses. Moreover, the multivariate analysis identified several risk factors for cardiovascular-pulmonary disease-related death, including male sex, earlier year of diagnosis, and squamous cell carcinoma pathologic type.

These appeared as unexpected findings. This study failed to show a difference in cardiovascular-pulmonary disease mortality, perhaps due to the follow-up not being long enough for outcomes to occur. The median durations of follow-up were 29 months in the Non-PORT group and 28.5 months in the PORT group, respectively. Cardiac-pulmonary toxicity following radiotherapy has been observed in long-term breast cancer and Hodgkin lymphoma survivors, with a typical latency period of more than a decade and increased incidence at younger age at treatment [18, 21, 24]. A previous study evaluating breast cancer after RT showed that the risk of major cardiovascular disease events started within the first 5 years after treatment and continued into the third decade of follow-up [29]. These malignancies portend a more favorable prognosis, while lung cancer is usually associated with poor prognosis and is conversely the leading cause of cancer-related death in the world [1]. In the phase III Lung ART trial [9], the 3-year OS rates were 66.5% in the PORT arm and 68.5% in the observation arm, respectively. In this study, NSCLC was the leading cause of death, with a cumulative incidence rate of 67.37%. It is possible that there was insufficient time for the development of cardiovascular-pulmonary disease in patients with stage IIIA-N2 NSCLC.

The definitive role of PORT in cardiopulmonary toxicity in pIIIA-N2 NSCLC remains controversial. In the recent PORT-C trial [12], no RT-induced grade 4 or 5 adverse events were observed. A total of 97 deaths (26.6%) occurred; among the 8 noncancer-related deaths, only 3 (3.1%) were due to cardiopulmonary disease [12]. The findings of the Lung ART study differed from the previous abovementioned study [9], and cardiopulmonary-specific mortality was observed in 2 (2.0%) patients in the Non-PORT cohort and 16 (16.2%) patients in the PORT cohort, respectively. The main reasons for these inconsistent results lie in the discrepancy of radiotherapy techniques and dose restrictions to the heart and lung. Studies have confirmed that patients treated with intensity-modulated radiation therapy (IMRT) for locally advanced NSCLC had lower rates of severe pneumonitis and cardiac doses than those treated with three-dimensional conformal external beam radiation therapy (3D-CRT) [30, 31]. The majority of patients received IMRT (89.3%) in the PORT-C study and 3D-CRT (89%) in the Lung ART study, respectively. The planned delivered dose was 54 Gy/27-30f in the LUNG ART trial. In reality, however, the maximum irradiation dose reached 70 Gy. The lung V20 was limited to less than 31% in patients after lobectomy and 22% after pneumonectomy, heart V30 less than 35% [9]. Another explanation for the low rate of cardiopulmonary-specific mortality in the PORT-C study is the markedly tighter dose restrictions for normal healthy tissues, especially the lungs and heart [12]. More effective modern RT techniques might have attenuated this risk.

Although our study showed no association of PORT with an increased risk of cardiovascular-pulmonary death in patients with stage IIIA-N2 NSCLC, the long-term safety of PORT for those patients remains uncertain. Combined with the results from the two randomized phase III trials [9, 12], new and modern RT techniques such as the use of IMRT are expected to avoid organs at risk and thereby diminish toxicities [30, 31]. Future studies exploring the long-term effects of modern RT on cardiovascular-pulmonary disease morbidity and mortality in NSCLC are required.

Despite the meaningful insights into radiation-induced cardiovascular-pulmonary disease mortality in NSCLC patients, we acknowledge several limitations. First, this study was based on the SEER database with potential hidden biases. The adoption of PSM in this study balances baseline patient characteristics between groups. Second, the SEER database lacks related information on pre-existing cardiovascular risk factors and cardiovascular-pulmonary diseases, which might influence subsequent mortality. Finally, type of chemotherapy and other treatment modalities (such as immune checkpoint inhibitors) were not available in the SEER database, which is closely associated with cardiopulmonary toxicity.

## Conclusions

In conclusion, this study shows the cumulative incidence of cardiovascular-pulmonary mortality by PORT use in stage IIIA-N2 NSCLC patients after complete resection and identifies potential prognostic factors for cardiovascular-pulmonary-specific death. Moreover, no significant differences were found in cardiovascular-pulmonary‐related modalities between the PORT and Non‐PORT groups in this study. Further studies are needed to assess these results, and more detailed information on risk factors should be examined in future work. This study highlights the importance of long-term surveillance for NSCLC patients.

## Data Availability

Data files were downloaded directly from the SEER website.

## Abbreviations

PORT: Postoperative radiotherapy
NSCLC: Non-small cell lung cancer
SEER: Surveillance, epidemiology, and end results
PSM: Propensity score matching
HR: Hazard ratio
CI: Confidence interval
OS: Overall survival
RT: Radiotherapy
LN: Lymph nodes
IMRT: Intensity-modulated radiation therapy
3D-CRT: Three-dimensional conformal external beam radiation therapy
